# Supplementary material for: Open structure and gating of the Arabidopsis mechanosensitive ion channel MSL10
Source: Nat Commun. 2023 Oct 7;14:6284. doi: 10.1038/s41467-023-42117-5 (PMC10560256; doi:10.1038/s41467-023-42117-5)
Supplement: Supplementary file 1 — Supplementary Information [file 41467_2023_42117_MOESM1_ESM.pdf]

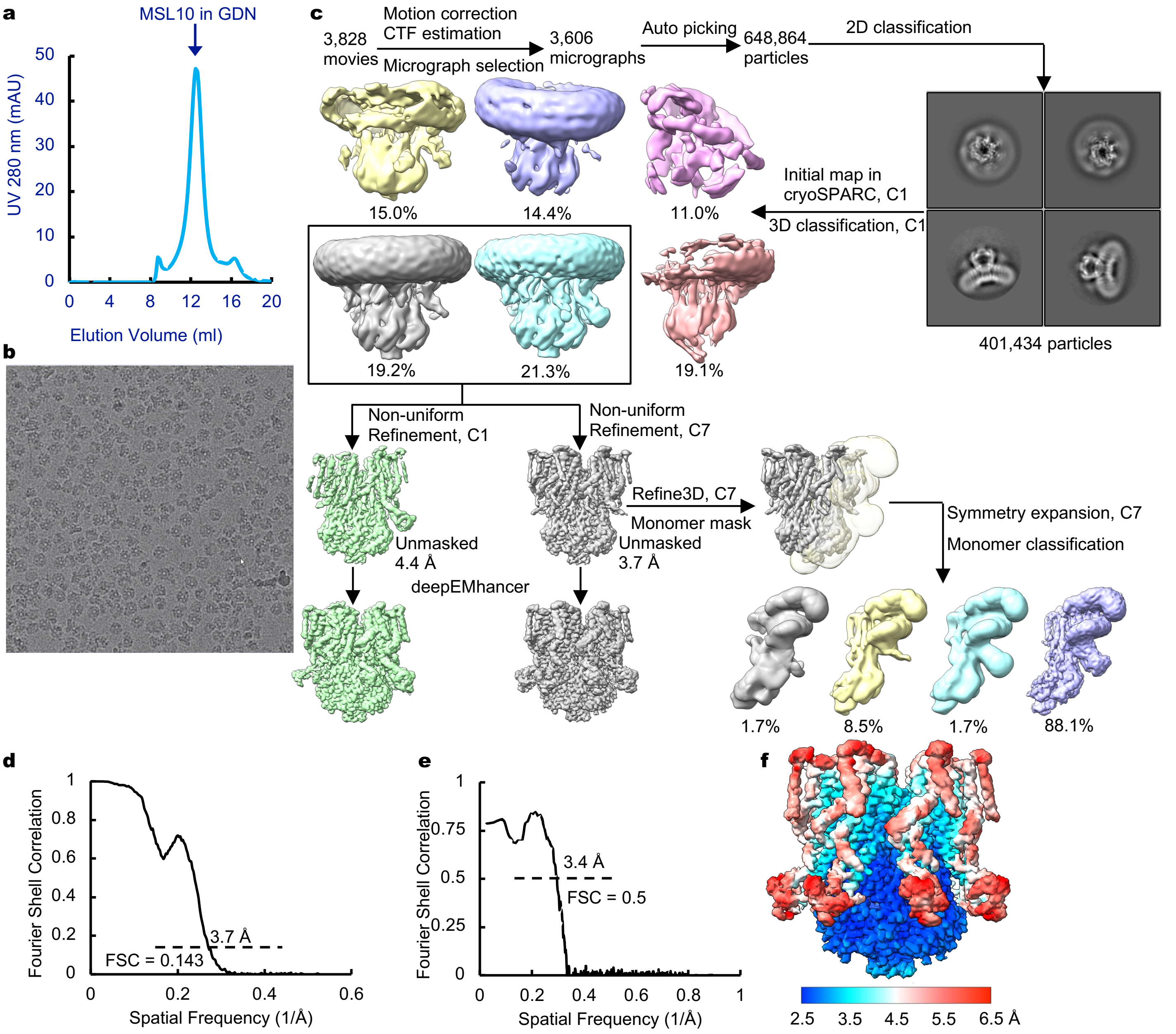

Supplementary Fig. 1 | Cryo-EM studies of *At*MSL10. a, Size-exclusion chromatography of the full-length wild-type *At*MSL10 purified in GDN for single-particle cryo-EM analysis. **b**, Representative micrograph. **c**, Flowchart of image processing. **d**, Fourier shell correlation calculated in cryoSPARC between half maps. **e**, Fourier shell correlation between the refined model and map. **f**, Local resolution plot of the map sharpened by deepEMhancer.

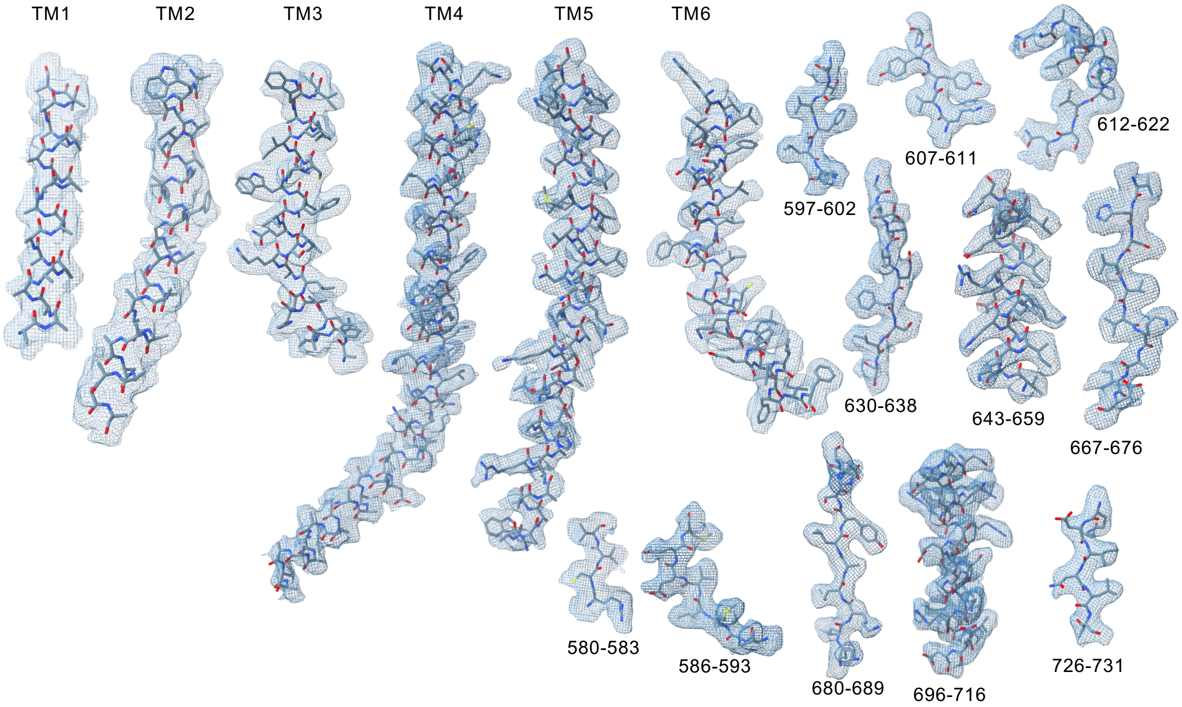

Supplementary Fig. 2 | Cryo-EM density of the wild-type full-length *At*MSL10 in GDN. The cryo-EM map was sharpened with a B-factor of -144.4. Also shown is the final refined atomic model.

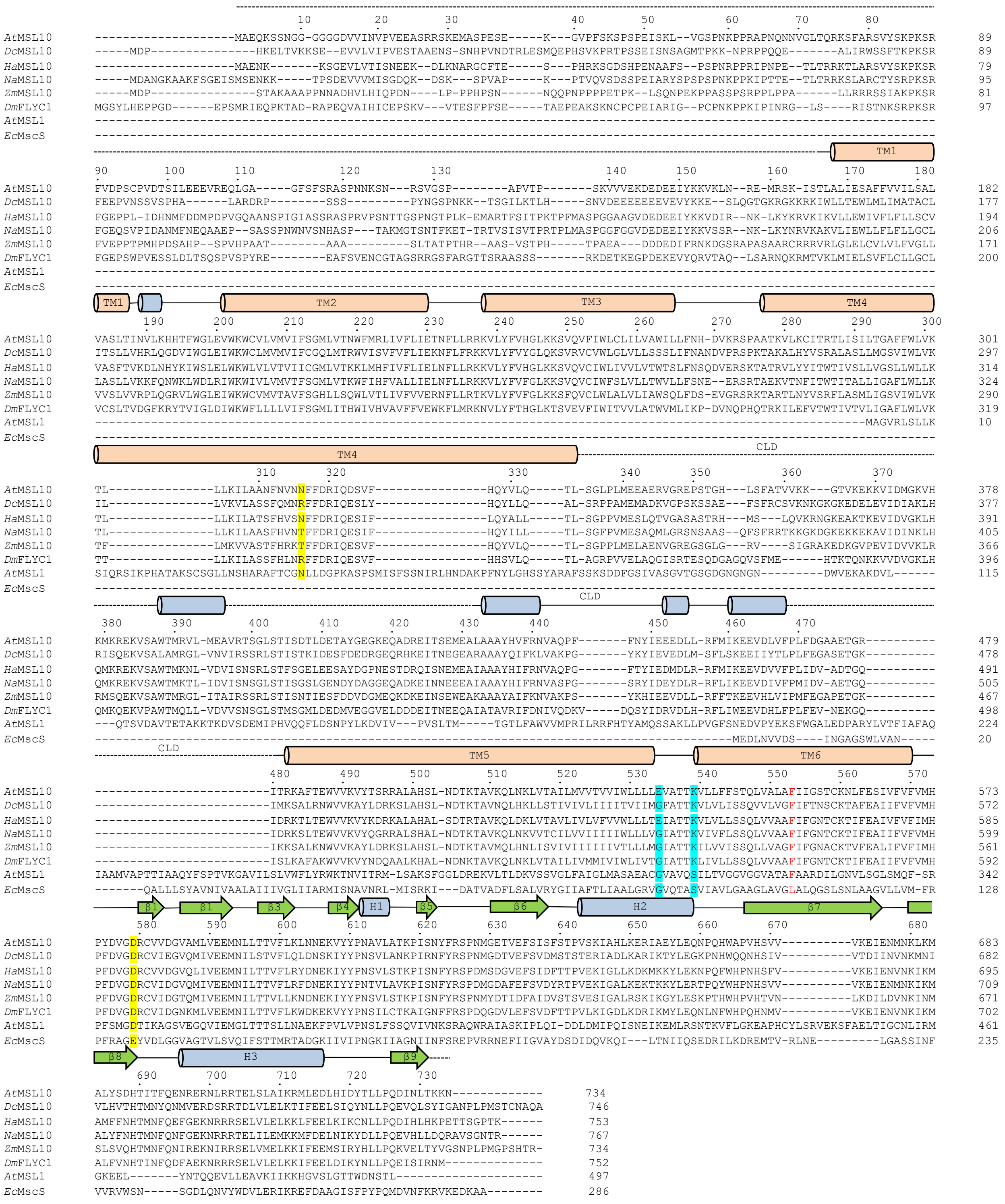

Supplementary Fig. 3 | Sequence alignment of MSL10 homologs. Sequences of *At*MSL10 (NCBI: NP_001119212.1), *Dc*MSL10 (*Dendrobium catenatum*, NCBI: PKU77895.1), *Ha*MSL10 (*Helianthus annuus*, NCBI: XP_021989332.1), *Na*MSL10 (*Nicotiana attenuate*, NCBI: XP_019229549.1), *Zm*MSL10 (*Zea mays*, NCBI: PWZ17953.1), *Dm*FLYC1 (*Dionaea muscipula*, NCBI: QNN26181.1), *At*MSL1 (NCBI: NP_567165.2) and *Ec*MscS (NCBI: NP_417399.1). Secondary structure elements of *At*MSL10 are shown above the sequences. The dotted line indicates the unresolved region in the cryo-EM reconstruction. Critical residues N316, E534, K539, F553, and D579 are highlighted.

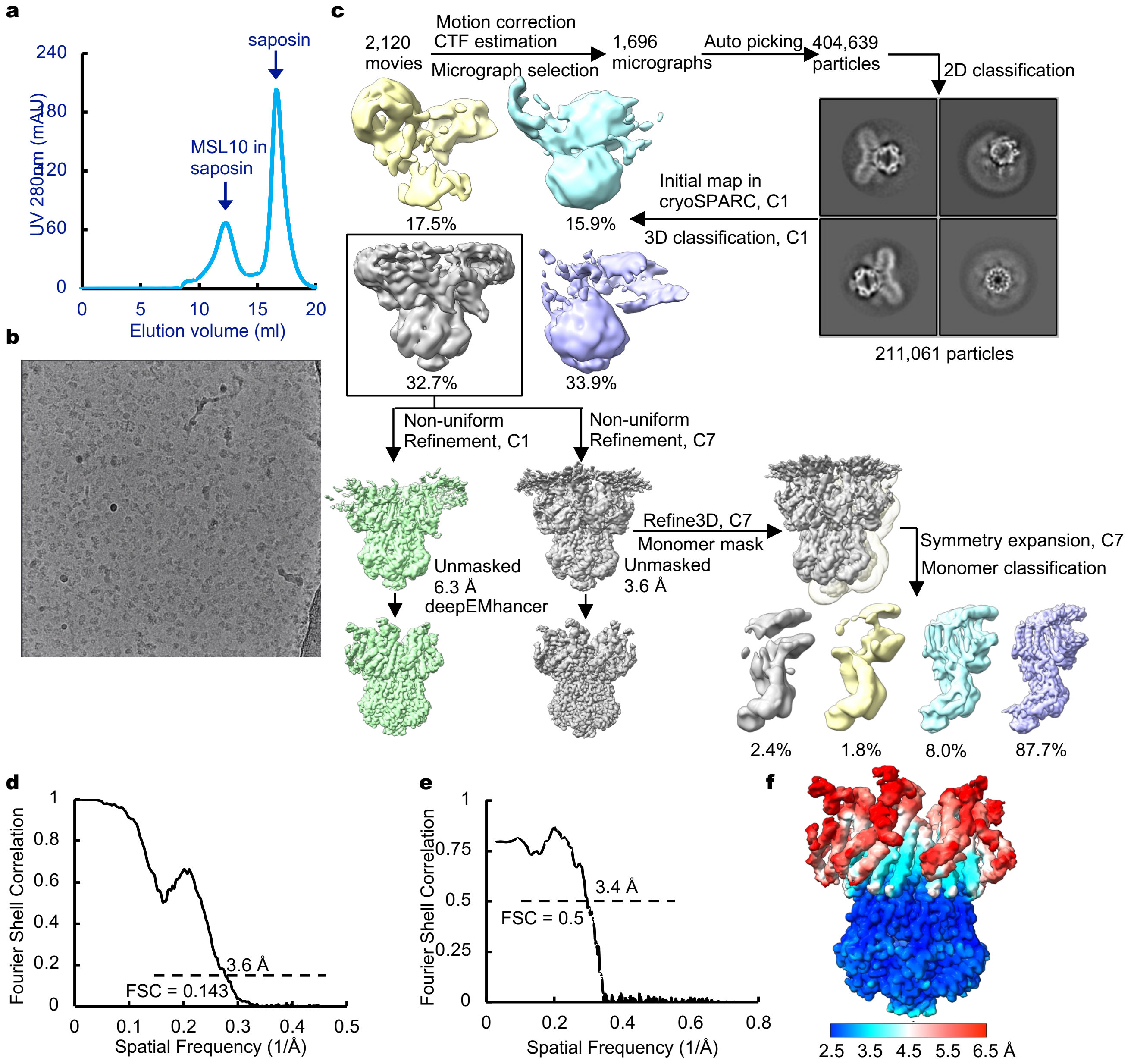

Supplementary Fig. 4 | Cryo-EM analysis of *At*MSL10 in saposin lipid nanoparticles. **a**, Size-exclusion chromatography of the full-length wild-type *At*MSL10 reconstituted in saposin lipid nanoparticles. **b**, Representative micrograph of *At*MSL10 in saposin lipid nanoparticles. **c**, Workflow of image processing. **d**, Fourier shell correlation between half maps. **e**, Fourier shell correlation between the final refined model and map. **f**, Local resolution plot of the map sharpened by deepEMhancer.

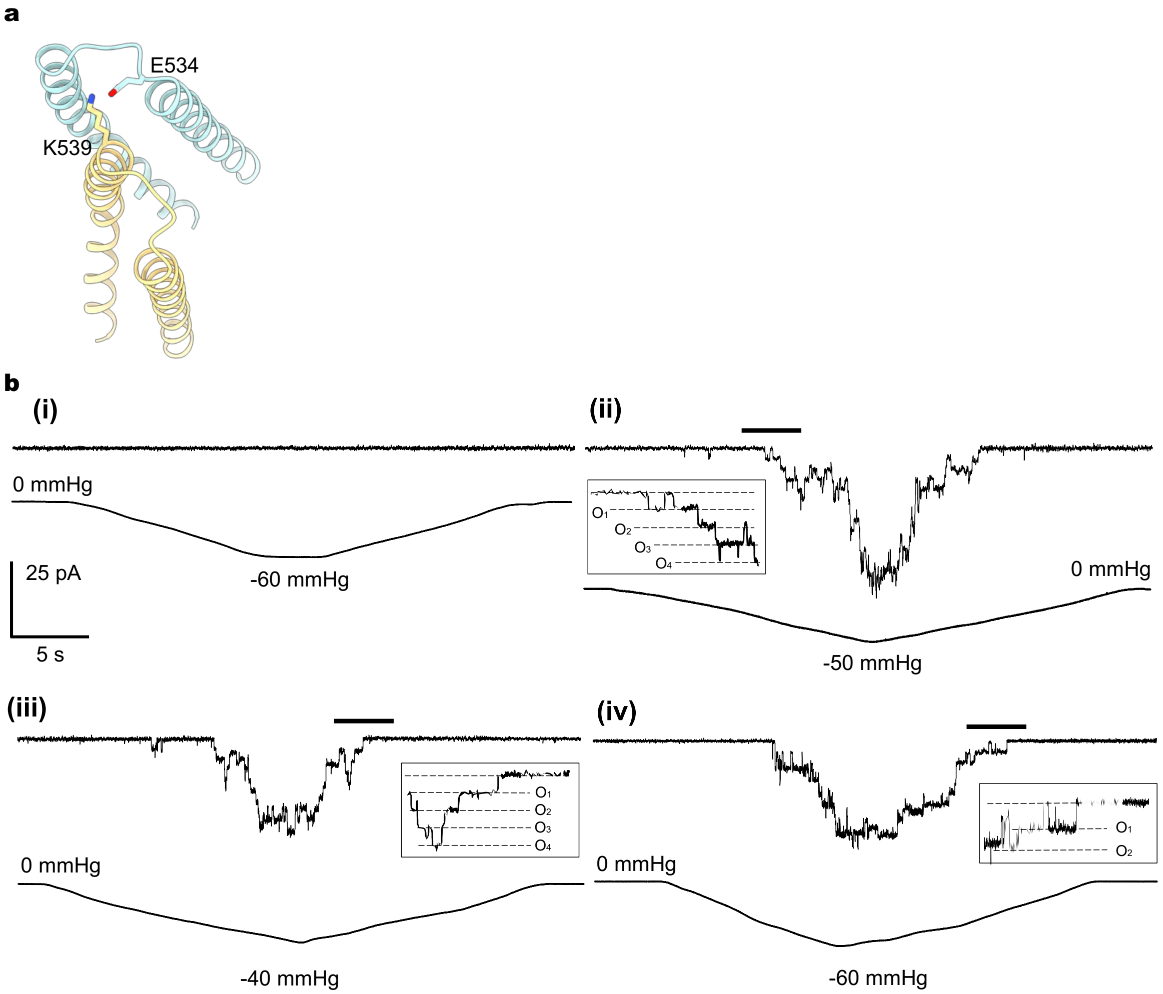

Supplementary Fig. 5 | The E534-K539 salt bridge. a, E534 in the TM5-TM6 linker from one subunit and K539 in TM6 from an adjacent subunit are highlighted in stick representation. **b**, Representative current traces of the wild-type *At*MSL10 and its mutants in excised membrane patches with applied pressure: (i) control, (ii) wild type, (iii) E534K, and (iv) K539E. The membrane potential is hold at -40 mV.

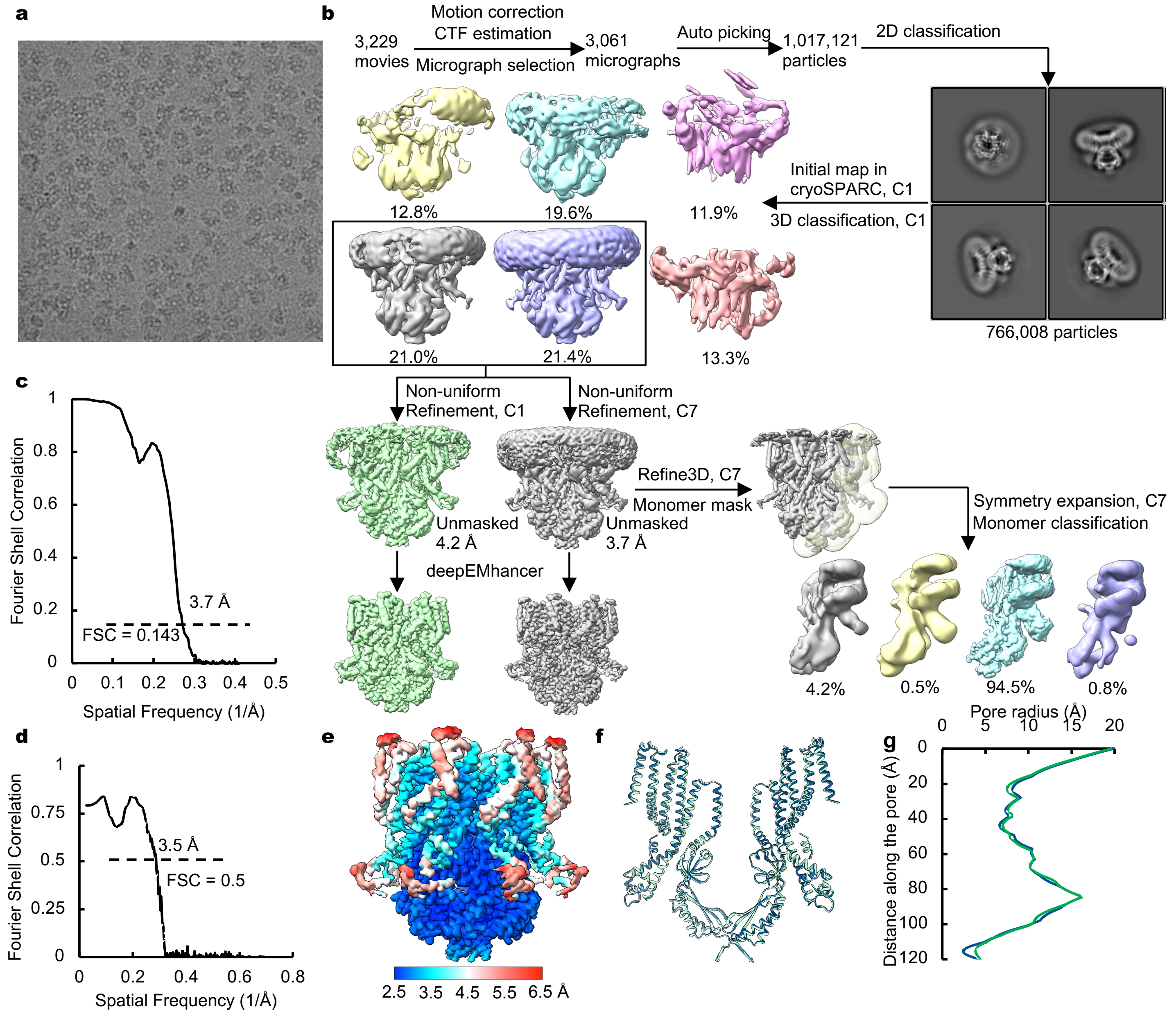

Supplementary Fig. 6 | Cryo-EM analysis of *At*MSL10 K539E. **a**, Representative micrograph. **b**, Workflow of image processing. **c**, Fourier shell correlation between half maps. **d**, Fourier shell correlation between the final refined model and map. **e**, Local resolution plot of the map sharpened by deepEMhancer. **f**, Superposition of structures of K539E (green) and the wild type (blue). **g**, Comparison of the pore profiles of the wild type (blue) and K539E (green).

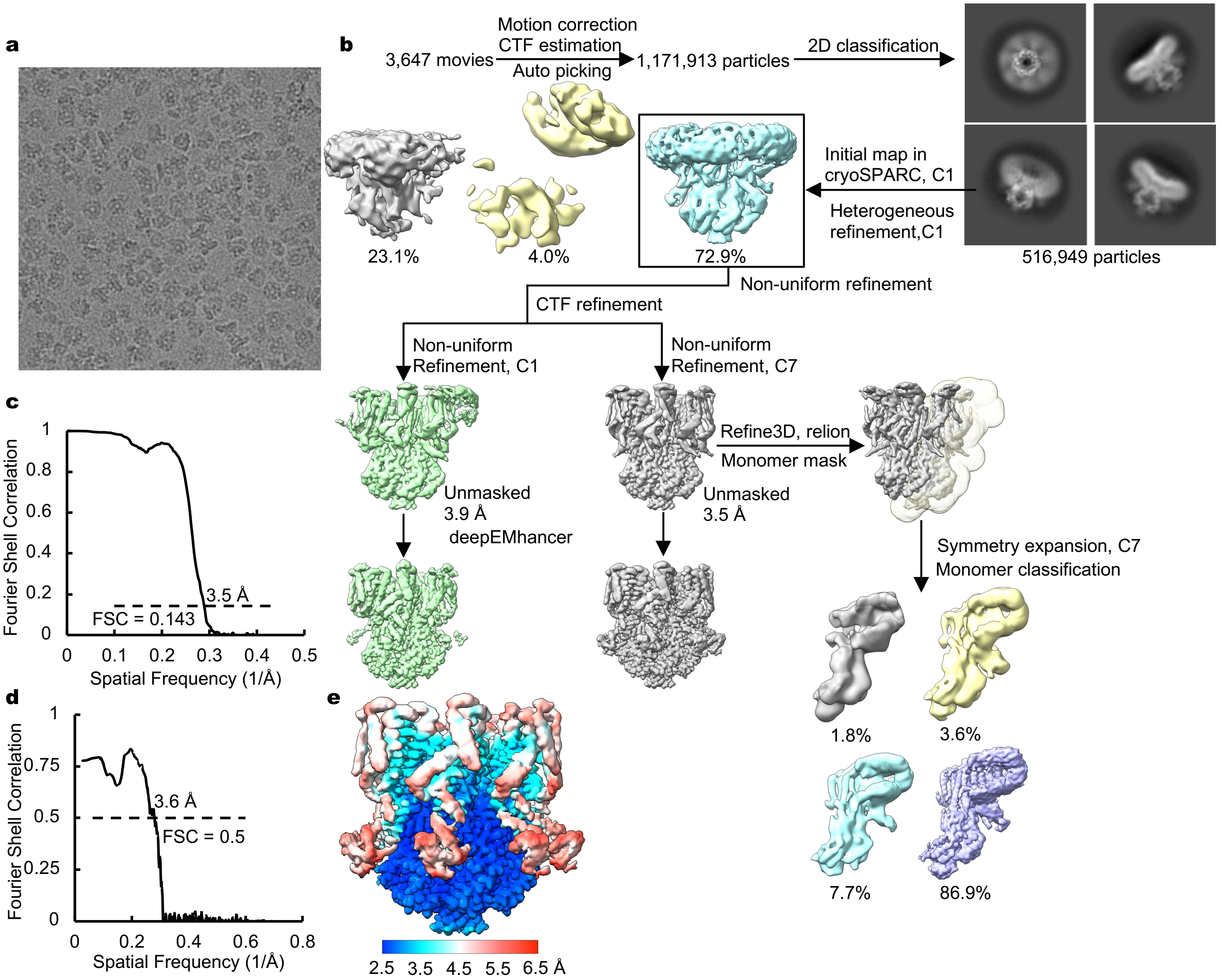

Supplementary Fig. 7 | Cryo-EM analysis of *At*MSL10 G556V. a, Representative micrograph. **b**, Workflow of image processing. **c**, Fourier shell correlation between half maps. **d**, Fourier shell correlation between the final refined model and map. **e**, Local resolution plot of the map sharpened by deepEMhancer.

Supplementary Table 1 | Cryo-EM data collection, refinement and validation statistics

	*At*MSL10 in detergent (EMD-41164) (PDB 8TDJ)	*At*MSL10 in saposin lipid nanoparticles (EMD-41166) (PDB 8TDL)	*At*MSL10 K539E (EMD-41168) (PDB 8TDM)	*At*MSL10 G556V (EMD-41165) (PDB 8TDK)
**Data collection and processing**				
Magnification	150k	59k	120k	120k
Voltage (kV)	200	300	200	200
Electron exposure (e^{-}/Å^2)	53	54	44	44
Defocus range (μm)	-0.8 to -2.4	-0.8 to -2.4	-0.8 to -2.4	-0.8 to -2.4
Pixel size (Å)	0.94	1.1	1.2	1.2
Symmetry imposed	C7	C7	C7	C7
Initial particle images (no.)	648,864	404,639	1,017,121	1,171,913
Final particle images (no.)	163,661	69,275	326,262	376,738
Map resolution (Å)	3.7	3.6	3.7	3.5
FSC threshold	0.143	0.143	0.143	0.143
Map resolution range (Å)	2.5-6.5	2.5-6.5	2.5-6.5	2.5-6.5
**Refinement**				
Initial model used (PDB code)	AlphaFold	This study	This study	This study
Model resolution (Å)	3.4	3.4	3.5	3.6
FSC threshold	0.5	0.5	0.5	0.5
Map sharpening *B* factor (Å^2)	-144.4	-50	-184.1	-50
Model composition				
Nonhydrogen atoms	22,771	17,535	22,750	22,274
Protein residues	3,269	2,548	3,269	3,269
Ligands	0	0	0	0
B factors (Å^2)				
Protein	145.76	159.35	156.76	180.48
Ligand	N/A	N/A	N/A	N/A
R.m.s. deviations				
Bond lengths (Å)	0.002	0.003	0.002	0.002
Bond angles (°)	0.464	0.487	0.436	0.425
Validation				
MolProbity score	1.12	1.24	1.24	1.24
Clash score	3.29	4.65	4.74	4.71
Poor rotamers (%)	0.36	0	0	0
Ramachandran plot				
Favored (%)	99.35	98.88	98.26	98.04
Allowed (%)	0.65	1.12	1.74	1.96
Disallowed (%)	0	0	0	0

Supplementary Table 2 | Primers used for cloning

Construct	Primer	Sequence (5′-3′)
*At*MSL10 E539K	forward	AAAGTTGCTACCACCAAAG
	reverse	CAACAACAACAACCAGATAAC
*At*MSL10 K539E	forward	GAAGTTTTGTTGTTTTTCTCTACC
	reverse	GGTGGTAGCAACTTCC
*At*MSL10 G556V	forward	TTTCTACCTGTAAAAACCTG
	reverse	CAATAATAAAAGCCAAGGC
