# Supplementary material for: Open structure and gating of the Arabidopsis mechanosensitive ion channel MSL10
Source: Nat Commun. 2023 Oct 7;14:6284. doi: 10.1038/s41467-023-42117-5 (PMC10560256; doi:10.1038/s41467-023-42117-5)
Supplement: Supplementary file 3 — Reporting Summary [file 41467_2023_42117_MOESM3_ESM.pdf]

nature portfolio

Corresponding author(s): Peng Yuan

Last updated by author(s): Sep 26, 2023

Reporting Summary

Nature Portfolio wishes to improve the reproducibility of the work that we publish. This form provides structure for consistency and transparency in reporting. For further information on Nature Portfolio policies, see our Editorial Policies and the Editorial Policy Checklist.

Statistics

For all statistical analyses, confirm that the following items are present in the figure legend, table legend, main text, or Methods section.

n/a	Confirmed	
☐	☒	The exact sample size (*n*) for each experimental group/condition, given as a discrete number and unit of measurement
☐	☒	A statement on whether measurements were taken from distinct samples or whether the same sample was measured repeatedly
☒	☐	The statistical test(s) used AND whether they are one- or two-sided<br>*Only common tests should be described solely by name; describe more complex techniques in the Methods section.*
☒	☐	A description of all covariates tested
☒	☐	A description of any assumptions or corrections, such as tests of normality and adjustment for multiple comparisons
☒	☐	A full description of the statistical parameters including central tendency (e.g. means) or other basic estimates (e.g. regression coefficient) AND variation (e.g. standard deviation) or associated estimates of uncertainty (e.g. confidence intervals)
☒	☐	For null hypothesis testing, the test statistic (e.g. *F*, *t*, *r*) with confidence intervals, effect sizes, degrees of freedom and *P* value noted<br>*Give P values as exact values whenever suitable.*
☒	☐	For Bayesian analysis, information on the choice of priors and Markov chain Monte Carlo settings
☒	☐	For hierarchical and complex designs, identification of the appropriate level for tests and full reporting of outcomes
☒	☐	Estimates of effect sizes (e.g. Cohen's *d*, Pearson's *r*), indicating how they were calculated

Our web collection on statistics for biologists contains articles on many of the points above.

Software and code

Policy information about availability of computer code

Data collection	EPU 2 (ThermoFisher Scientific), Axopatch 1D patch-clamp amplifier, Digidata 1320 digitizer (Molecular Devices), PM-015R pressure monitor (World Precision Instruments), pClamp software suite 8.2 (Molecular Devices)
Data analysis	pClamp software suite 10.7 (Molecular Devices), cryoSPARC v4.2.1, Relion 3.1.2, MotionCor2_1.4.0, GCTF_1.06, DeepEMhancer 0.14, COOT 0.9, Phenix 1.19.2 and 1.20.1, ChimeraX 1.5, Chimera 1.14, HOLE 2.2.005

For manuscripts utilizing custom algorithms or software that are central to the research but not yet described in published literature, software must be made available to editors and reviewers. We strongly encourage code deposition in a community repository (e.g. GitHub). See the Nature Portfolio guidelines for submitting code & software for further information.

Data

Policy information about availability of data

All manuscripts must include a data availability statement. This statement should provide the following information, where applicable:

- Accession codes, unique identifiers, or web links for publicly available datasets
- A description of any restrictions on data availability
- For clinical datasets or third party data, please ensure that the statement adheres to our policy

The cryo-EM maps have been deposited to Electron Microscopy Data Bank with accession codes EMD-41164 (https://www.ebi.ac.uk/pdbe/entry/emdb/ EMD-41164), EMD-41165 (https://www.ebi.ac.uk/pdbe/entry/emdb/ EMD-41165), EMD-41166 (https://www.ebi.ac.uk/pdbe/entry/emdb/ EMD-41166), and EMD-41168 (https://www.ebi.ac.uk/pdbe/entry/emdb/ EMD-41168). Atomic coordinates have been deposited to the Protein Data Bank (PDB) with accession codes

8TDJ (https://www.rcsb.org/structure/ 8TDJ), 8TDK (https://www.rcsb.org/structure/ 8TDK), 8TDL (https://www.rcsb.org/structure/ 8TDL), and 8TDM (https://www.rcsb.org/structure/8TDM). Correspondence and requests for materials should be addressed to P.Y. (peng.yuan@mssm.edu).

Research involving human participants, their data, or biological material

Policy information about studies with human participants or human data. See also policy information about sex, gender (identity/presentation), and sexual orientation and race, ethnicity and racism.

Reporting on sex and gender	N/A
Reporting on race, ethnicity, or other socially relevant groupings	N/A
Population characteristics	N/A
Recruitment	N/A
Ethics oversight	N/A

Note that full information on the approval of the study protocol must also be provided in the manuscript.

Field-specific reporting

Please select the one below that is the best fit for your research. If you are not sure, read the appropriate sections before making your selection.

☒ Life sciences ☐ Behavioural & social sciences ☐ Ecological, evolutionary & environmental sciences

For a reference copy of the document with all sections, see nature.com/documents/nr-reporting-summary-flat.pdf

Life sciences study design

All studies must disclose on these points even when the disclosure is negative.

Sample size	3,828 micrographs for the wild-type AtMSL10 in GDN, 2,120 micrographs for the wild-type AtMSL10 in saposin, 3,229 micrographs for AtMSL10 K539E, and 3,647 micrographs for AtMSL10 G556V were collected. These sample sizes are sufficient for 3D reconstructions.
Data exclusions	For cryo-EM reconstruction of the wild-type AtMSL10 in GDN, 648,864 particles were included in 2D and 3D classification. 485,203 particles resulted in partial protein density or low resolution in 2D or 3D classification were discarded.<br>For cryo-EM reconstruction of the wild-type AtMSL10 in saposin, 404,639 particles were included in 2D and 3D classification. 335,364 particles resulted in partial protein density or low resolution in 2D or 3D classification were discarded.<br>For cryo-EM reconstruction of AtMSL10 K539E, 1,017,121 particles were included in 2D and 3D classification. 690,859 particles resulted in partial protein density or low resolution in 2D or 3D classification were discarded.<br>For cryo-EM reconstruction of AtMSL10 G556V, 1,171,913 particles were included in 2D and 3D classification. 795,175 particles resulted in partial protein density or low resolution in 2D or 3D classification were discarded.
Replication	Electrophysiological experiments were duplicated. Results are reproducible.
Randomization	For electrophysiology, the cells expressing the AtMSL10 channel and its mutants were randomly selected. Cryo-EM particles were randomly separated into two half-sets to generate the half maps.
Blinding	The investigators were blinded to group allocation during data collection and analysis.

Reporting for specific materials, systems and methods

We require information from authors about some types of materials, experimental systems and methods used in many studies. Here, indicate whether each material, system or method listed is relevant to your study. If you are not sure if a list item applies to your research, read the appropriate section before selecting a response.

Materials & experimental systems

n/a	Involved in the study
☒	☐ Antibodies
☐	☒ Eukaryotic cell lines
☒	☐ Palaeontology and archaeology
☒	☐ Animals and other organisms
☒	☐ Clinical data
☒	☐ Dual use research of concern
☒	☐ Plants

Methods

n/a	Involved in the study
☒	☐ ChIP-seq
☒	☐ Flow cytometry
☒	☐ MRI-based neuroimaging

Eukaryotic cell lines

Policy information about cell lines and Sex and Gender in Research

Cell line source(s)	Pichia pastoris SMD1163H
Authentication	N/A
Mycoplasma contamination	Cells were not tested for mycoplasma contamination
Commonly misidentified lines (See ICLAC register)	N/A
